# Anion Binding Studies of Urea and Thiourea Functionalized Molecular Clefts

**DOI:** 10.3389/fchem.2020.575701

**Published:** 2021-01-29

**Authors:** Utsab Manna, Bobby Portis, Tochukwu K. Egboluche, Muhammad Nafis, Md. Alamgir Hossain

**Affiliations:** Department of Chemistry, Physics and Atmospheric Sciences, Jackson State University, Jackson, MS, United States

**Keywords:** molecular cleft, urea, thiourea, anion binding, chemosensor

## Abstract

Two rationally designed 4-nitrophenyl-based molecular clefts functionalized with thiourea (**L**_**1**_) and urea (**L**_**2**_) have been synthesized and studied for a variety of anions by UV-Vis and colorimetric techniques in DMSO. Results from the binding studies suggest that both **L**_**1**_ and **L**_**2**_ bind halides showing the order: fluoride > chloride > bromide > iodide; and oxoanions showing the order: dihydrogen phosphate > hydrogen sulfate > nitrate > perchlorate. Each receptor has been shown to form a 1:1 complex with an anion *via* hydrogen bonding interactions, displaying distinct color change for fluoride and dihydrogen phosphate in solution. As compared to the urea-based receptor **L**_**2**_, the thiourea-based receptor **L**_**1**_ exhibits stronger affinity for anions due the presence of more acidic thiourea functional groups.

## Introduction

Because of the fundamental role played by anions in biological, industrial, and environmental processes, selective binding and sensing of anions by synthetic receptors has become one of the most extensively studied areas in supramolecular chemistry (Sessler et al., 2008; Bowman-James, 2005). As anions are vital in a wide range of chemical research such as functional materials, transmembrane transports, and catalysis, the anion binding chemistry has been proven to be an immense area of research interest to the scientific community (Wiskur et al., 2001; Martinez-Manez et al., 2003; Gale et al., 2008; Caltagirone et al., 2009; Gale et al., 2010; Wenzel et al., 2012).

Among the various anions, halides and oxoanions have a great impact on both human health and environment. For example, an elevated amount of fluoride in drinking water causes dental and skeletal fluorosis (Ayoob and Gupta, 2006). Fluoride has an adverse effect in human body, resulting in a bone cancer known as *osteosarcoma* (Ayoob and Gupta, 2006). Sulfate and phosphate anions are known to bind to proteins in the neutral environment of respective sulfate and phosphate binding proteins (Pflugrath and Quiocho, 1985; Wang et al., 1997). Dihydrogen phosphate becomes the most abundant species among the three forms of inorganic phosphates (H_2_PO_4_^−^, HPO_4_^2−^ and PO_4_^3−^) and shows donor-acceptor properties similar to those of the water molecule. Hence, the recognition and detection of these key inorganic anions by efficient artificial sensors is much anticipated. In a recent report, it was shown that C_3v_-symmetric receptors possessing both H-bond donor and acceptor exhibited high selectivity for H_2_PO_4−_ over other common inorganic anions (Park et al., 2016; Kim, et al., 2018; Kim, et al., 2019). It is now documented that a urea or thiourea group is capable to chelate an anion *via* two directional H-bonds (Jia et al., 2016). Therefore, the molecules containing (thio)urea moieties with flexible (Nishizawa et al., 1995; Nishizawa et al., 1998; Emgenbroich et al., 2008; Duke et al., 2012; Caltagirone et al., 2013; Olivari et al., 2015; Gillen et al., 2018) or rigid (Manna et al., 2016; Manna and Das 2017; Khansari et al., 2017b; Manna et al., 2017; Gale and Gunnlaugsson, 2010) linkers have been successfully applied as a promising class of receptors for anion recognition purpose in recent past years. Nishizawa et al. (1995) reported bis(thio)urea receptors containing *n*-butyl terminals with a flexible *meta*-xylylenediamine-based linker, exhibiting preferable binding for H_2_PO_4_^−^ and SO_4_^2−^ over other anions as evaluated by NMR studies. The same group also synthesized phenyl-substituted ureas as an ionophore for sulfate selective electrode (Nishizawa et al., 1998). Emgenbroich et al. (2008) synthesized a *meta*-xylylenediamine-based vinylphenyl urea, showing good interactions for tyrosine phosphorylated peptides. Caltagirone et al. (2013) described pyrophosphate optical sensing properties of *meta*-xylylenediamine derivatives bearing naphthalene and 2-nitrobenzene moieties. Further studies on similar types of receptors indicated that such receptors can be useful for selective anion transports (Olivari et al., 2015). Li et al. (2013) reported *ortho*-phenylene connected *bis*-thio(urea) receptors exhibiting interactions with oxoanions, while both bisurea and thiourea were shown to form *tris*-chelate complexes with phosphate ion (PO_4_^3−^) in the solid state. C_3v_-symmetric tripodal receptors with various flexible and rigid frameworks have been shown to exhibit for the chromogenic and fluorogenic sensing of anions through the formation of hydrogen bonded host-guest complex followed by the deprotonation of amide-NH protons (Kim et al., 2015; Kim et al., 2016; Sahoo et al., 2016).

**SCHEME 1:**
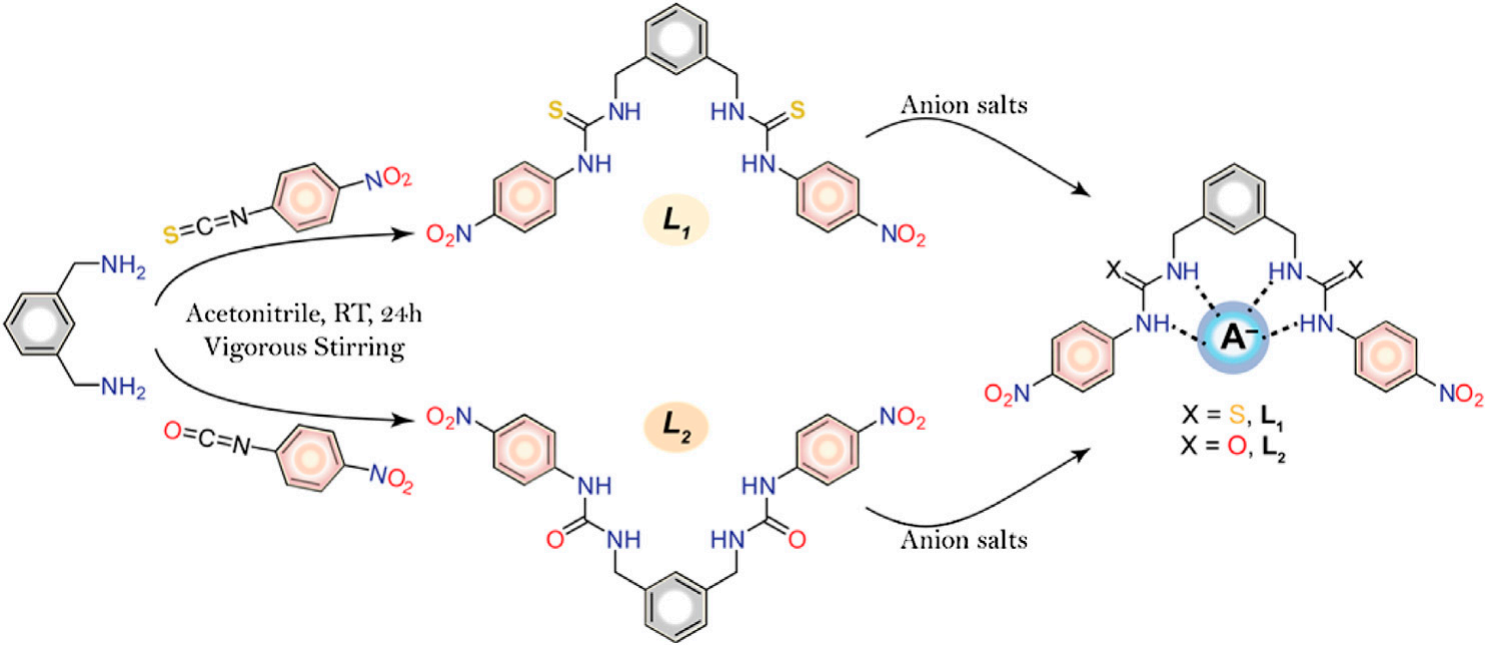
Synthetic route of the receptors and schematic diagram of anion-receptor binding through probable H-bonding interactions.

In an effort to develop new colorimetric chemosensors for naked eye detection of anions (Basaran et al., 2014; Haque et al., 2015; Rhaman et al., 2018; Rhaman et al., 2020; Portis et al., 2020), we have synthesized two simple *meta*-xylylene-based molecular clefts: one containing thiourea (**L**_**1**_) and other containing urea (**L**_**2**_) functionalities (Scheme 1). Herein we report the synthesis of these receptors and their binding behavior for halides and oxoanions, which exhibit preferential binding as well as visible color change for fluoride and phosphate in DMSO. Due to the presence of more acidic thiourea groups in **L**_**1**_ as compared to its urea analogue **L**_**2**_, the former exhibits stronger interactions for anions than its counterpart.

## Material and Methods

### Materials

All reagents and solvents were purchased from commercial suppliers and used without further purification. Nuclear magnetic resonance (NMR) spectra were recorded at 25 °C on a Varian Unity INOVA 500 FT-NMR instrument and the chemical shifts were recorded in parts per million (ppm) using tetramethylsilane as a reference. All NMR data were processed and analyzed with MestReNova software. The following abbreviations in ^1^H NMR characterization are used to describe spin multiplicities: s = singlet; d = doublet; t = triplet; and m = multiplet. The absorption spectra for UV-Vis screening and titrations were performed in DMSO at room temperature using a UV-2600 spectrophotometer (SHIMADZU). Elemental analysis was carried out using an ECS 4010 Analytical Platform (Costech Instrument) at Jackson State University.

### Synthesis

The receptors were successfully synthesized (Scheme 1) by the reaction of *m*-xylylenediamine (0.5 ml, 3.7 mmol) with 4-nitrophenyl isothiocyanate (1.3 g, 7.7 mmol) for **L**_**1**_ or 4-nitrophenyl isocyanate (1.2 g, 7.7 mmol) for and **L**_**2**_ in dry acetonitrile under constant stirring at room temperature. After overnight stirring, the yellowish precipitate was filtered off and washed with acetonitrile and THF. The residues were dried under vacuum to give **L**_**1**_ (88% yield) and **L**_**2**_ (92% yield).

**L**_**1**_: ^1^H NMR (500 MHz, DMSO-*d*_*6*_) *δ* (ppm): 4.77 (s, 4H, xylyl–C*H*_2_), 5.76 (s, 1H, Ar–*H*), 7.26–7.27 (d, 2H, ∼7.5 Hz, Ar–*H*), 7.33–7.36 (t, 1H, ∼7.0 Hz, Ar–*H*), 7.82–7.84 (d, 4H, ∼9.0 Hz, Ar–*H*), 8.15 (d, 4H, ∼9.0 Hz, Ar–*H*), 8.71 (s, 2H, N*H*_*a*_), 10.26 (s, 2H, N*H*_*b*_).

^13^C NMR (125 MHz, DMSO-*d*_*6*_) *δ* (ppm): 47.5 (xylyl–*C*H_2_), 120.0 (Ar–*C*), 124.9 (Ar–*C*), 126.9 (Ar–C), 128.9 (Ar–*C*), 138.9 (Ar–*C*), 180.7 (*C*=S). IR (KBr, ν/cm^−1^): 3,334, 3,190, 2,930, 1,594, 1,533, 1,506, 1,326, 1,303, 1,245. Anal. Calcd. for C_22_H_20_N_6_O_4_S_2_: C, 53.21; H, 4.06; N, 16.92. Found: C, 53.25; H, 4.07; N, 16.88.

**L**_**2**_: ^1^H NMR (500 MHz, DMSO-*d*_*6*_) *δ* (ppm): 4.774 (s, 4H, Xylyl–CH_2_), 5.760 (s, 1H, Ar–H), 7.259–7.272 (d, 2H, ∼6.5 Hz, Ar–H), 7.335–7.356 (t, 1H, ∼6.0 Hz, Ar–H), 7.825–7.838 (d, 4H, ∼6.5 Hz, Ar–H), 8.149–8.162 (d, 4H, ∼6.5 Hz, Ar–H), 8.717 (s, 2H, NH_a_), 10.270 (s, 2H, NH_b_).

^13^C NMR (125 MHz, DMSO-*d*_*6*_) *δ* (ppm): 44.5 (xylyl–*C*H_2_), 118.2.0 (Ar–*C*), 125.69 (Ar–*C*), 128.0 (Ar–C), 141.8 (Ar–*C*), 146.2 (Ar–*C*) 152.2 (*C*=S). IR (KBr, ν/cm^−1^): 3360, 3340, 2,940, 1734, 1,594, 1,495, 1,250, 1,207, 1,177. Anal. Calcd. for C_22_H_20_N_6_O_6_: C, 56.89; H, 4.34; N, 18.10. Found: C, 56.85; H, 4.37; N, 18.08.

### UV-Vis Titration Studies

Stock solutions of various anions (1 × 10^−1^ M) and receptors (5 × 10^−3^ M) were prepared separately in DMSO. In order to obtain an optimal absorption, the solutions of **L**_**1**_ and **L**_**2**_ were diluted to 2.5 × 10^−5^ M. In the UV-Vis screening experiment, the samples were prepared by mixing 10 equivalents of the stock solutions of different anions into a quartz optical cell of 1 cm path length filled with 2.0 ml of individual receptor solution. In a typical titration experiment, UV abortion spectra were recorded from the gradual addition (up to 35 equivalents) of a respective anionic solution (2.5 × 10^−3^ M) to **L1** or **L2**. The receptor-anion binding constant values (log *K*) were determined from UV-Visible titration studies using a 1:1 binding model (Hynes 1993). The error limit was less than 10%.

## Results and Discussion

### Synthetic Design and Binding Studies

The synthesis of molecular clefts **L**_**1**_ and **L**_**2**_ was accomplished in a single-step reaction (Scheme 1) as a pure form, providing a good yield (88–92%). The principle of designing a chemosensor is based on the fundamental features that a probe within a single framework should possess anion binding sites for recognizing a particular anion through noncovalent interactions and suitable chromophores for providing a desired absorption band at a UV-Vis wavelength. We also attempted to study the anion binding behavior of these receptors using NMR titration studies. However, the addition of fluoride or phosphate resulted into the broadening of NH resonances, thus hampering the determination of binding constants. For other anions, NMR resonances were not significant enough to calculate the binding constants.

### UV-Vis Spectroscopic Studies

The presence of two nitrophenyl units as chromophores in both receptors **L**_**1**_ and **L**_**2**_ with anion binding sites, allowed us to use them as potential colorimetric probes for anions. Earlier work has proven that the nitrophenyl groups functionalized to certain receptors could act as effective chromophores for anion sensing (Jia et al., 2016; Khansari et al., 2017a). Hence, extensive UV-Vis screening experiments and colorimetric studies were performed to examine the sensitivity of the receptors for anions. Furthermore, the binding interactions of the probes for different anions were evaluated by UV-Vis titration experiments in DMSO.

**FIGURE 1:**
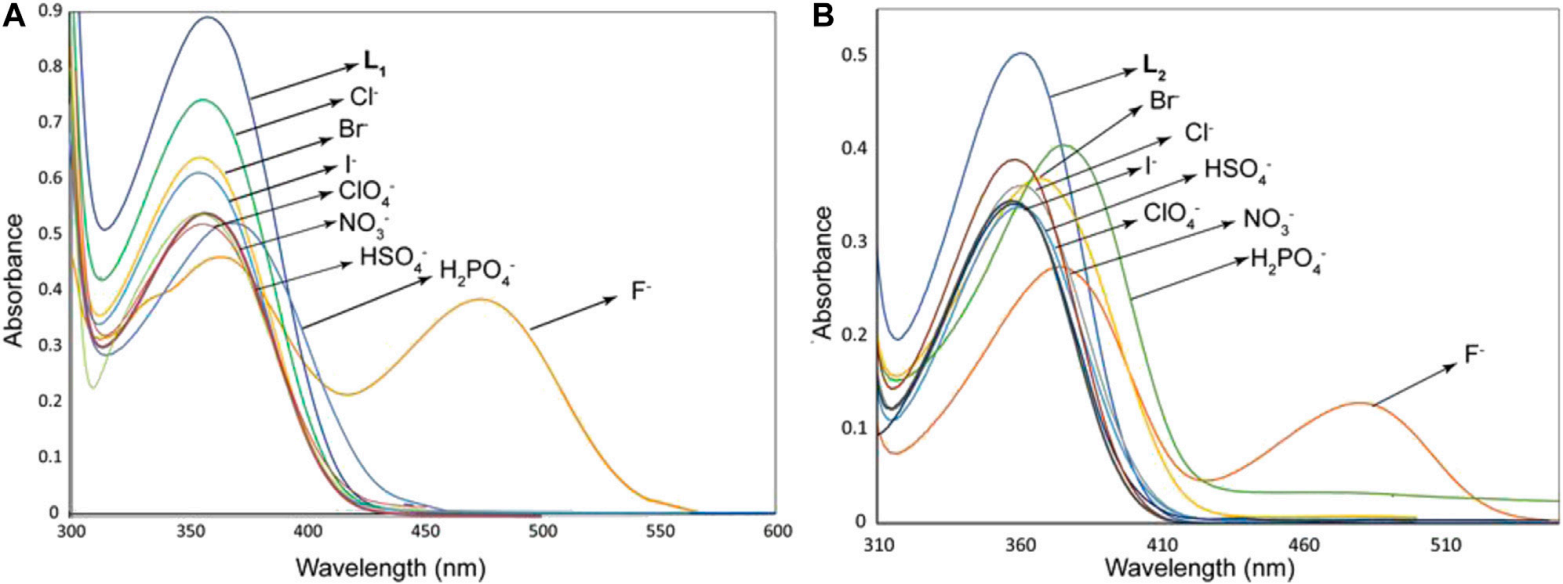
UV-Vis screening experiments of **L**_**1**_ (2.5 × 10^−5^ M) **(A)** and **L**_**2**_ (2.5 × 10^−5^ M) **(B)** with different anions (1.5 × 10^−3^ M) in DMSO, showing changes in the absorption bands.

**FIGURE 2:**
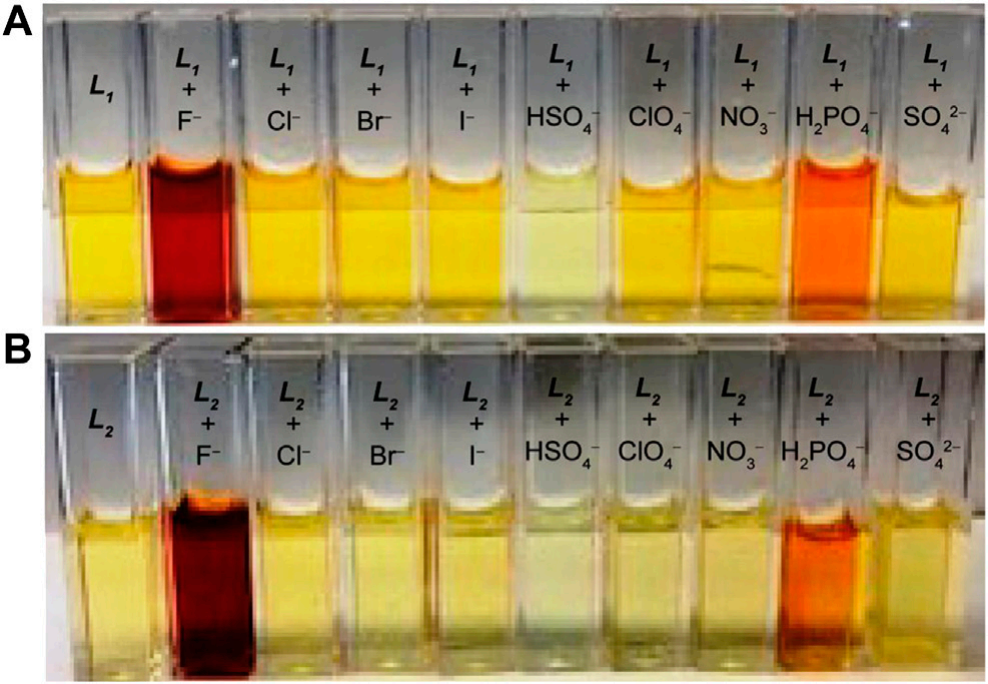
Colorimetric studies of **(A) L**_**1**_ and **(B) L**_**2**_ in the presence of different anions in DMSO, showing a color change for fluoride and dihydrogen phosphate ([host]_0_ = 2 × 10^−3^ M, [anion]_0_/[host]_0_ = 10).

As shown in Figure 1, the probes **L**_**1**_ and **L**_**2**_ in their free states showed intense bands at 355 and 357 nm, respectively in DMSO. After the addition of different anions (10 equivalents) including F^−^, Cl^−^, Br^−^, I^−^, HSO_4_^−^, H_2_PO_4_^−^, NO_3_^−^ and ClO_4_^−^ as their salts of [*n-*Bu_4_N]^+^ to the probes, we observed remarkable spectral changes of **L**_**1**_ and **L**_**2**_ in terms of both intensity and wavelength with fluoride and dihydrogen phosphate, as compared to other anions included in our study. This observation suggests that these two anions (fluoride and dihydrogen phosphate) are capable to induce a colorimetric response in DMSO due to the formation of a strong anion complex. The corresponding colorimetric experiments, as performed with the addition of 10 equivalents of the respective anions to **L**_**1**_ and **L**_**2**_ (2 × 10^−3^ M) are shown in Figure 2, displaying a sharp color change for fluoride (dark red) and dihydrogen phosphate (red), agreeing with the results of UV-Vis screening studies.

**FIGURE 3:**
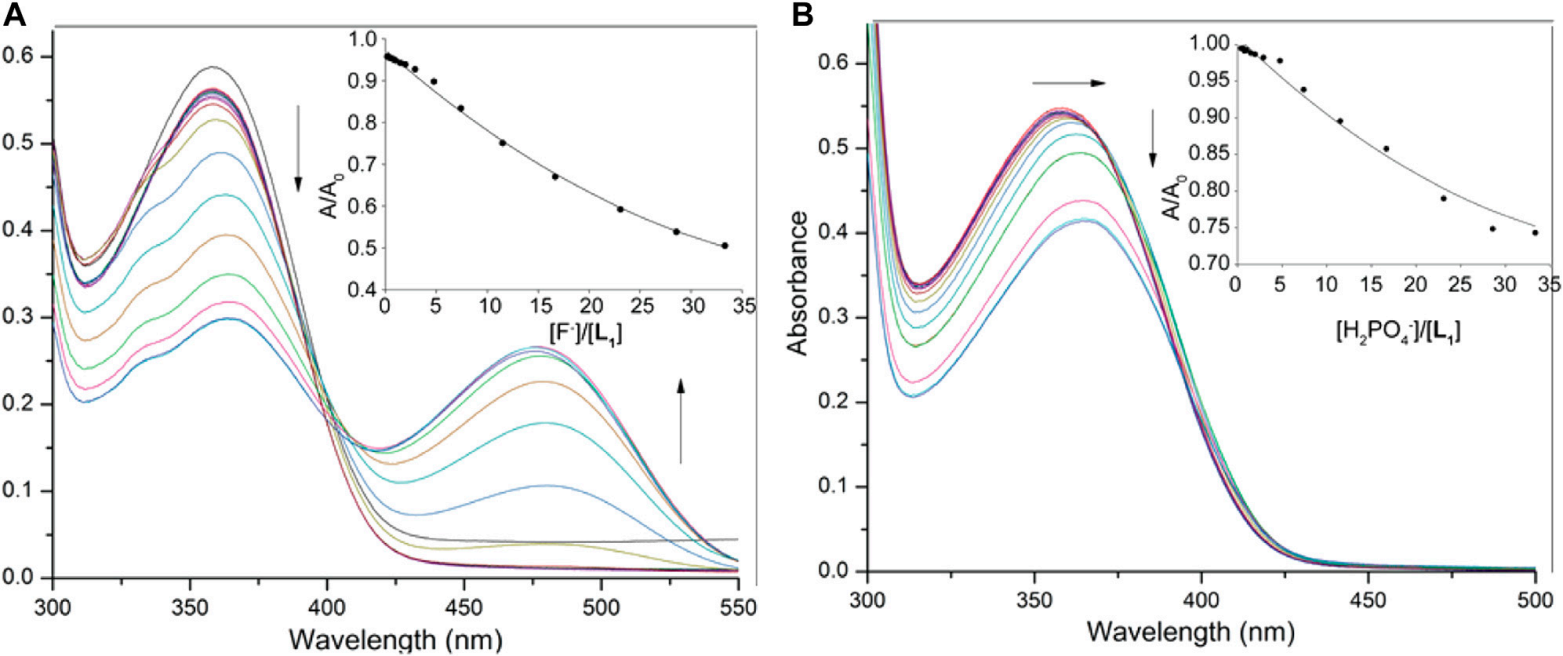
UV-Vis titration spectra of **L**_**1**_ (2.5 × 10^−5^ M) in DMSO with varying concentration of **(A)** fluoride (1.5 × 10^−3^ M), and **(B)** dihydrogen phosphate (1.5 × 10^−3^ M); titration fitting curve showing changes in the absorbance with incremental addition of F^−^ (at *λ*_max_ = 355 nm) and H_2_PO_4_^−^ (at *λ*_max_ = 358 nm) anions are shown in insets.

The anion binding strengths of the receptors were evaluated by UV-Vis titration studies in DMSO. The titration profile of **L**_**1**_ with fluoride, as shown in Figure 3A, demonstrates a systematic decrease (hypochromic shift) in the absorption band at 355 nm with a red shift (Δλ_max_ = 6–7 nm), yielding an isosbestic point at around 395 nm. A new peak appears in the visible range at around 478 nm, showing an increase in intensity (hyperchromic shift) with the concentration of fluoride, suggesting the formation of a receptor-fluoride complex. This spectral change is consistent with the visual color change (from pale yellow to deep red, Figure 2A) due to the complex formation, offering the scope for the naked eye detection of fluoride in solution. The relative absorbance *I*/*I*_*0*_ of the peak at 355 nm (where, *I*_*0*_ and *I* represent the absorbance of **L**_**1**_ before and after the addition of fluoride, respectively) due to the gradual addition of fluoride provided the best fit to a 1:1 binding mode, yielding a binding constant of 3.70 (in log *K*, Figure 3A). The acidic NH groups within the host molecule allow to bind F^−^ within the cavity of **L**_**1**_, thereby forming a [**L**_**1**_·F]^-^ complex through hydrogen-bonding interactions (Scheme 1). This binding constant is comparable to that reported previously with a dipodal thiourea (log *K* = 3.69, Khansari et al., 2014) or a tris(3-aminopropyl) amine-based tripodal thiourea (log *K* = 3.81, Khansari et al., 2015); however, somewhat lower than that with a tris(2-aminoethyl) amine-based tripodal thiourea (log *K* = 5.1, Khansari et al., 2017a).

On titration with dihydrogen phosphate, the receptor **L**_**1**_ showed a similar decrease in the absorption band (*λ*_max_ at 355 nm), while the peak was shifted to about *λ*_max_ at 363 nm at the end of the titration (Figure 3B). However, no new peak was observed on the visible range. The change of the relative absorbance *I*/*I*_*0*_ at *λ*_max_ at 355 nm was consistent with a 1:1 complex, providing a binding constant of log *K* = 2.82. A closely related rigid bis-thiourea receptor reported by Li et al. was shown to bind dihydrogen phosphate ion with higher affinity (log *K* = 3.49) may be because of more converging dipodal arms (Li et al., 2013). Continuing titrations with other anions including Cl^−^, Br^−^, I^−^, HSO_4_^−^, NO_3_^−^ and ClO_4_^−^ showed a systematic decrease in the absorption band (*λ*_max_ at 355 nm). Notably, no shifting of *λ*_max_ was found (Supplementary Material) which is consistent with the colorimetric results, displaying no apparent color change due to the addition of these anions to the receptor. The binding data (Table 1), as obtained from non-linear regression analysis (Hynes, 1993), suggest that the receptor binds strongly toward F^−^ anion (log *K* = 3.70). Under the same condition, it binds other anions (Br^−^, I^−^, HSO_4_^−^, NO_3_^−^ and ClO_4_^−^) weakly, agreeing with the reported binding trend for other neutral receptors (Khansari et al., 2017a). The higher affinity for F^−^ is due to the fact that this anion is more basic as compared to other anions, thereby forming strong anion-complexes with the acidic thiourea groups of **L**_**1**_ through H-bonding interactions.

**TABLE 1:** Binding constants of the receptors estimated from UV-Visible titration studies.

Anions	Binding constants (log *K*)	
	L_1_	L_2_
F^−^	3.70	2.84
Cl^−^	2.55	1.67
Br^−^	1.89	1.52
I^−^	1.54	1.41
H_2_PO_4_^−^	2.82	2.07
HSO_4_^−^	2.04	1.58
NO_3_^−^	1.44	1.40
ClO_4_^−^	1.50	1.45

**FIGURE 4:**
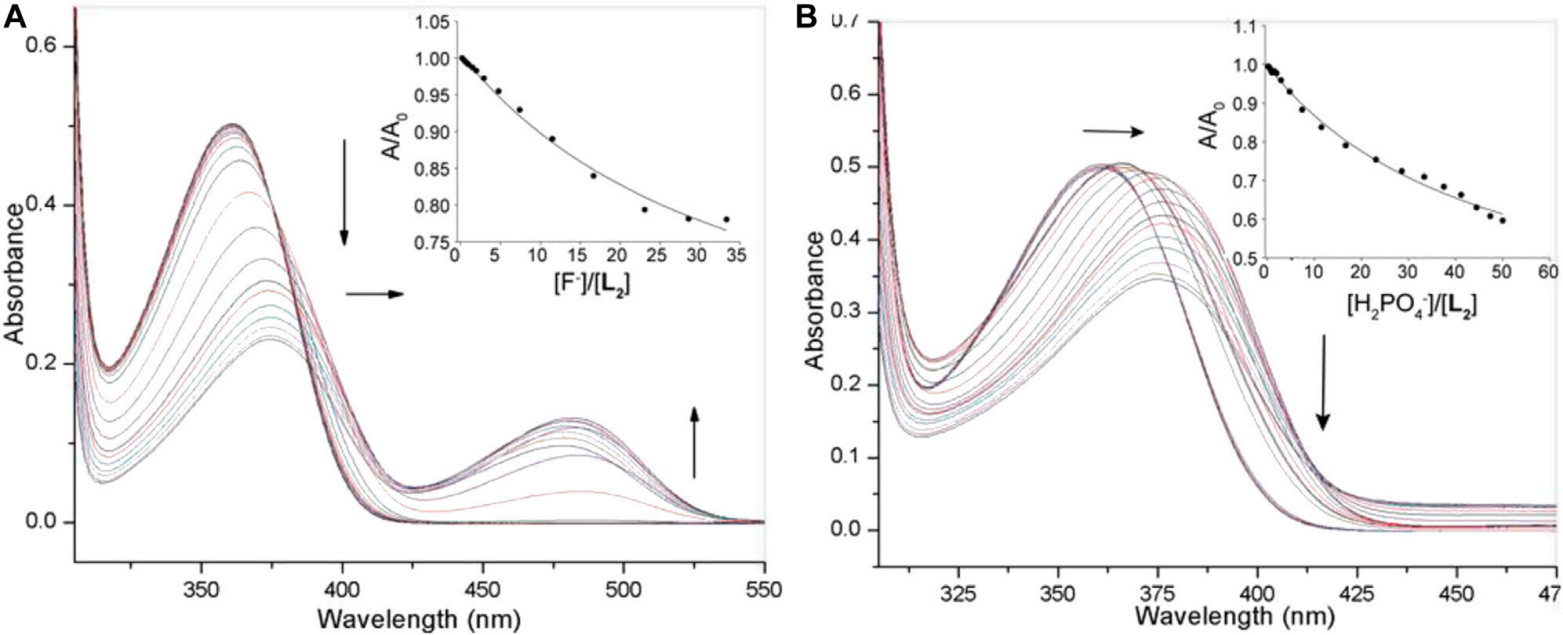
UV-Vis titration spectra of **L**_**2**_ (2.5 × 10^−5^ M) in DMSO with varying concentration of **(A)** fluoride (1.5 × 10^−3^ M), **(B)** dihydrogen phosphate (1.5 × 10^−3^ M); Titration fitting curve showing changes in the absorbance with the incremental addition of F^−^ (at *λ*_max_ = 355 nm) and H_2_PO_4_^−^ (at *λ*_max_ = 360 nm) anions are shown in insets.

In order to assess and compare the binding constants of the urea analogue **L**_**2**_, we also performed UV-Vis titrations with anions, showing almost similar spectral changes as observed in the case of **L**_**1**_ under the same conditions (Figure 4A). The titration profile of receptor **L**_**2**_ with fluoride revealed a systematic decrease in the absorption band (*λ*_max_) at 357 nm followed by a red shift (Δλ_max_ = 10–12 nm), while a new peak appeared at around 480 nm was shown to increase with the incremental addition of fluoride to the receptor. With dihydrogen phosphate, it showed a red shift (Δλ_max_ = 15 nm) of the absorption band (*λ*_max_) at 357 nm at the end of the titration (Figure 4B). The corresponding colorimetric results, showing visible color change are again in accord with these observations (Figure 2B). The binding constants, as listed in Table 1, clearly demonstrate that both receptors exhibited a similar trend of binding for anions, showing the higher affinity for F^−^ and H_2_PO_4_^−^ with a binding trend of F^−^ > H_2_PO_4_^−^ > Cl^−^ > HSO_4_^−^ > Br^−^ > I^−^ > ClO_4_^−^ > NO_3_^−^. It is noted that the thiourea-based receptor **L**_**1**_ shows an improved binding for an anion as compared to its urea analogue, accounting the enhanced acidity of NHs in **L**_**1**_ offered by the thiourea groups (Custelcean et al., 2005; Li et al., 2013).

## Conclusion

In summary, we have synthesized two simple dipodal urea/thiourea clefts; each appended on a *meta*-xylyl based molecular framework and investigated their affinities toward a variety of inorganic anions. The inclusion of a nitro group as a chromophore allows the molecules to use them as effective probes for spectroscopic and visual discrimination of fluoride (from light yellow to dark red) and dihydrogen phosphate (from light yellow to red) in solution. In particular, each of the receptors exhibited strong affinities for fluoride with the binding trend for halides in the order: fluoride > chloride > bromide > iodide; and for oxoanions in the order: dihydrogen phosphate > hydrogen sulfate > nitrate > perchlorate, forming 1:1 complexes with anions by virtue of hydrogen bonding interactions. The thiourea-based receptor as compared to its urea analogue, exhibits an increased affinity for anions due the presence of enhanced acidity of thiourea units. This study could serve as an insight for the rational design of new functional chemosensors for selective sensing of anions in an application oriented field.

## Data Availability Statement

The original contributions presented in the study are included in the article/Supplementary Material, further inquiries can be directed to the corresponding author.

## Author Contributions

MH conceived the project idea. UM, BP, and MN synthesized and studied the compounds. TE assisted in titration studies. UM prepared the initial manuscript. All authors approved the manuscript.

## Funding

The project described was supported by the US Department of Defense (Grant No. W911NF-19-1-0006). 

## Conflict of Interest

The authors declare that the research was conducted in the absence of any commercial or financial relationships that could be construed as a potential conflict of interest.
